# Efficacy of Bimekizumab on Palmoplantar Psoriasis: A 16-Week Multicenter Retrospective Study—IL PSO (Italian Landscape PSOriasis)

**DOI:** 10.3390/jcm15114168

**Published:** 2026-05-28

**Authors:** Martina Burlando, Lidia Sacchelli, Alberta Bettacchi, Giovanna Alexandra Brunasso Vernetti, Stefano Caccavale, Anna Balato, Anna Elisabetta Cagni, Karin Chersi, Andrea Conti, Antonio Costanzo, Domenico D’amico, Clara De Simone, Vito Di Lernia, Maria Esposito, Claudia Giofre’, Paolo Gisondi, Francesca Graziola, Serena Lembo, Matteo Licciardello, Francesco Loconsole, Piergiorgio Malagoli, Francesca Satolli, Maria Elena Susi, Davide Strippoli, Emanuele Claudio Cozzani

**Affiliations:** 1 Section of Dermatology, Department of Health Sciences (DISSAL), University of Genoa, 16132 Genova, Italy; martinaburlando@hotmail.com (M.B.);; 2 Section of Dermatology, IRCCS Azienda Ospedaliera Metropolitana, Ospedale San Martino, 16132 Genova, Italy; 3 Dermatology Unit, IRCCS Azienda Ospedaliero-Universitaria di Bologna, Policlinico S. Orsola Malpighi, 40138 Bologna, Italy; 4 Department of Medical and Surgical Sciences, Alma Mater Studiorum University of Bologna, 40126 Bologna, Italy; 5 UOC of Dermatology, Hospital of Macerata, ASUR Marche AV3, 62100 Macerata, Italy; alberta.bettacchi@sanita.marche.it; 6 Department of Dermatology, Galliera Hospital, 16128 Genova, Italy; alexandra.brunasso@galliera.it; 7 Unit of Dermatology, Nuovo Policlinico, Luigi Vanvitelli University of Campania, 80131 Naples, Italy; stefano.caccavale@unicampania.it (S.C.);; 8 Unità Operativa Dipartimentale di Dermatologia e Venereologia, IRCCS San Gerardo, 20900 Milan, Italy; 9 Dermatology Clinic, Hospital Clinics Giuliano Isontino (ASUGI), University of Trieste, 34127 Trieste, Italy; 10 Dermatologic Unit, Department of Surgery, Ospedale Infermi, AUSL Romagna, Viale Settembrini 2, 47923 Rimini, Italy; 11 Section of Dermatology, Department of Biomedical Sciences, Humanitas University, 20089 Rozzano, Italy; 12 Dermatology Unit, Hospital Facility “A. Pugliese”, UOC “R.Dulbecco”, 88100 Catanzaro, Italy; domenicodamico@live.it; 13 Department of Translational Medicine and Surgery, IRCCS A. Gemelli University Polyclinic Foundation, Sacred Heart Catholic University, 00168 Rome, Italy; 14 Dermatology Unit, Arcispedale Santa Maria Nuova, Azienda USL-IRCCS di Reggio Emilia, 42123 Reggio Emilia, Italy; vito.dilernia@ausl.re.it; 15 Department of Biotechnological and Applied Clinical Sciences, University of L’Aquila, 67100 L’Aquila, Italy; 16 U.O.C.di Dermatologia, Dermatology Unit, Azienda Ospedaliera Papardo, 98158 Messina, Italy; 17 Section of Dermatology, Department of Medicine, University of Verona, 37124 Verona, Italy; paolo.gisondi@univr.it; 18 SCDU Dermatologia, AOU Maggiore Della Carità, 28100 Novara, Italy; 19 Department of Medicine, Surgery and Dentistry, “Scuola Medica Salernitana”, University of Salerno, 84081 Salerno, Italy; slembo@unisa.it; 20 Section of Dermatology, Koelliker Hospital, 10134 Turin, Italy; 21 Dermatology Unit, Policlinico Consorziale, 70124 Bari, Italy; 22 Psocare Unit, IRCCS Policlinico San Donato, 20097 San Donato Milanese, Italy; 23 Unit of Dermatology, University of Parma, 43126 Parma, Italy; 24 Department of Health Sciences (DISSAL), University of Genoa, 16132 Genoa, Italy; 25 Dermatology Unit, Manzoni Hospital, ASST-Lecco, 23900 Lecco, Italy

**Keywords:** palmoplantar psoriasis, bimekizumab, psoriasis, difficult-to-treat areas, itch

## Abstract

**Background/Objectives**: Palmoplantar psoriasis (PP) is a challenging variant of psoriasis that affects high-impact areas such as palms and soles and significantly impairs quality of life despite often limited body surface involvement. Conventional topical and systemic therapies may be insufficient, and evidence on biologic treatments for this specific phenotype remains limited. Bimekizumab (BKZ), a monoclonal antibody targeting IL-17A and IL-17F, has shown high efficacy in plaque psoriasis. This study aimed to evaluate the real-world effectiveness and rapidity of action of BKZ in patients with palmoplantar psoriasis compared with patients with psoriasis vulgaris (PV). **Methods**: We conducted a multicenter retrospective cohort study using data from 22 Italian dermatological units within the IL-PSO (Italian Landscape-Psoriasis) database. Adult patients treated with BKZ between November 2022 and October 2024 were included and categorized into three groups: isolated PP, PP associated with PV (PP + PV), and PV without palmoplantar involvement. Clinical outcomes included the Psoriasis Area and Severity Index (PASI), Dermatology Life Quality Index (DLQI), and pruritus Visual Analogue Scale (VAS). Outcomes were assessed at baseline, week 4, and week 16. **Results**: A total of 47 patients were included. The baseline PASI was lower in the PP group compared with the PP + PV and PV groups, whereas the DLQI was highest in patients with isolated PP. Rapid clinical improvement was observed in all groups. The mean % PASI reduction at week 4 was 60.5%, 65.1%, and 77.0% in the PP, PP + PV, and PV groups, respectively, increasing to 94.8%, 90.4%, and 91.8% at week 16. The proportion of patients achieving complete clearance (PASI = 0) at week 16 was 73.3% (11/15), 68.2% (15/22), and 70.0% (7/10), respectively. Significant improvements were also observed in DLQI and pruritus scores over time. No significant safety concerns emerged. **Conclusions**: In this real-world multicenter cohort, bimekizumab demonstrated rapid and high efficacy in patients with palmoplantar psoriasis, both isolated and associated with psoriasis vulgaris. These findings support the use of BKZ as an effective therapeutic option for psoriasis involving high-impact areas, as the palmoplantar, although larger studies are needed to confirm these results.

## 1. Introduction

Palmoplantar psoriasis (PP) is a form of psoriasis that affects the palms and soles and can also involve so-called special sites or high-impact areas, such as the nails, scalp, and genital regions [1].

Up to 40% of patients with psoriasis can present PP alone or in association with psoriasis vulgaris (PV) at other body sites [2]. Usually, patients with PP alone, with limited psoriasis extension and a BSA < 10, are treated with topical therapies containing calcipotriol/betamethasone, which are considered first-line, followed by traditional systemic therapies, such as acitretin [1,3].

However, the palms and soles have thicker skin, which can make topical therapies ineffective or burdensome to apply, reducing patients’ compliance [1]. Furthermore, biotechnological therapies, which are very effective for PV, may show only mild efficacy in this form of psoriasis, especially compared to anti-TNF-a [4].

Moreover, involvement of the palms and soles in psoriasis is frequently associated with symptoms such as itching, which may interfere with daily activities and significantly impair quality of life [5].

Currently, no head-to-head trials are comparing the efficacy of available biologics in these difficult-to-treat areas. Some network meta-analyses are available and support bimekizumab (BKZ) as a valid option for an effective and rapid response [6].

Indeed, according to current knowledge, IL-17 plays a pivotal role in psoriasis pathogenesis by promoting angiogenesis, stimulating keratinocyte proliferation, and reducing apoptosis [7]. Specifically, several studies have shown that IL-17A is highly expressed in PP, whereas in plaque psoriasis, its expression is variable, ranging from intermittent to strong [8,9].

Given that PP represents a therapeutic challenge for physicians, we aimed to evaluate the effects in term of PASI and rapidity of action of bimekizumab (BKZ) in PP signs and symptoms in clinical practice collecting data from 23 Italian dermatological units.

## 2. Materials and Methods

This is a multicenter retrospective cohort study based on data collected from 22 Italian dermatological units (IL-PSO, Italian Landscape-Psoriasis database). Biotechnological therapies were administered according to the Italian adaptation of the EuroGuiDerm guidelines for the treatment of moderate-to-severe psoriasis [10]. Patient information was collected from November 2022 to October 2024. All data were anonymized and handled in accordance with local ethical regulations and the Declaration of Helsinki.

Inclusion criteria:

- Adult patients of both sexes
- A confirmed diagnosis of palmoplantar psoriasis, either isolated (PP cohort) or in association with other psoriatic manifestations (PP + PV cohort) OR a confirmed diagnosis of psoriasis vulgaris without palmoplantar involvement (PV cohort, comparative group).

Diagnosis was clinical, and dermoscopy was used as confirmation. No biopsies were performed.

- Treatment with bimekizumab;
- Bio-naive or bio-experienced status;
- Availability of complete clinical follow-up data, including baseline and post-treatment assessments until week 16 (W16);
- Use of topical therapies (calcipotriol/betamethasone) cream/ointment of gel was possible as add on therapy.

Exclusion criteria:

- Presence of other concomitant dermatological diseases that could interfere with PASI and PGA assessment;
- Incomplete follow-up data;
- Pregnancy, breastfeeding;
- Concomitant infections (HIV, HBV, HCV, or positive Quantiferon).

The following variables were recorded:

- Age, sex, and disease duration;
- Presence of additional psoriatic manifestations (onychopathy, scalp involvement, genital psoriasis, and psoriatic arthritis);
- Baseline and follow-up (W16) Psoriasis Area and Severity Index (PASI) scores, Dermatology Life Quality Index (DLQI);
- Pruritus assessment using the Visual Analogue Scale (VAS);
- Previous therapies undergone.

Bimekizumab was administered according to the standard dosing regimen.

The initial phase was as follows: 2 subcutaneous injections of 160 mg at weeks 0, 4, 8, 12, and 16.

The maintenance phase was as follows: 2 subcutaneous injections of 160 mg every 8 weeks.

### Statistical Analysis

Continuous variables were reported as the mean ± standard deviation (SD) and median with the interquartile range (IQR). Differences between baseline and follow-up time points were analyzed using paired *t*-tests for normally distributed variables and Wilcoxon signed-rank tests for non-normally distributed variables. Comparisons among the three groups were conducted using one-way ANOVA or a Kruskal–Wallis test, as appropriate. Categorical variables were analyzed using Fisher’s exact tests. A *p*-value < 0.05 was considered statistically significant. No correction for multiple comparisons was applied; all *p*-values were nominal and exploratory. Statistical analyses were performed using R version 4.3.1: A language and environment for statistical computing. R Foundation for Statistical Computing, Vienna, Austria.

This study was conducted in compliance with ethical guidelines, and the relevant institutional review boards approved data collection. Written informed consent was obtained from all patients prior to data entry into the database.

## 3. Results

A total of 47 consecutive adult patients with moderate-to-severe psoriasis were enrolled (Table 1). Males were 59.6% (28), and the mean age was 58.2 years (females: 58.04; males: 58.36); the mean disease duration was 14.4 years. Three patients out of 50 were excluded as they were lost at follow-up.

### 3.1. The Patients Were Divided into Three Groups

The first group, consisting of 15 patients out of 47 (32%), had only palmo-plantar psoriasis (PP) and is referred to as the pure PP group.

The second group, made up of 22 patients out of 47 (47%), had both palmoplantar psoriasis (PP) and psoriasis localized in other areas; this group is called the PP + PV group.

The third group included 10 patients out of 47 (21%) with other forms of psoriasis, known as the PV group.

In the PP group, there were 11 males (73.3%) and 4 females (26.7%), with a mean age of 57.3 years. In the PP + PV group, there were 12 males (54.5%) and 10 females (45.5%), with a mean age of 58.4 years. In the PV group, there were 5 males (50%) and 5 females (50%), with a mean age of 60.7 years. Sex distribution was comparable across the three groups (Fisher’s exact test: *p* = 0.408). Age was similarly distributed (Kruskal–Wallis test: H = 0.063, *p* = 0.969).

Regarding body mass index (BMI), the mean BMI in the pure PP group was 26.8 ± 3.7 kg/m^2^, in the PP + PV group, it was 27.1 ± 5.6 kg/m^2^, and in the PV group, it was 30.6 ± 4.9 kg/m^2^ (*p*-value = 0.045) (Table 2).

### 3.2. Bio-Naïve and Bio-Experienced

In all three groups, there were both treatment-naïve patients and patients previously exposed to biological therapies other than BKZ.

In the PP group, 7 patients (46.7%) were naïve, 6 (40%) had received another biologic besides BKZ, and 2 (13%) had used more than two biologic.

In the PP + PV group, 8 patients (36.4%) were naïve, 11 (50%) had previously used another biologic, and 3 (13.6%) had used more than two.

In the PV group, 3 patients (30%) were naïve, 4 (40%) had received another biologic, and 3 (30%) had used more than two.

### 3.3. PASI, DLQI, and VAS Itch Analysis

In the PP group, the mean baseline PASI before starting BKZ was 7.7, whereas in the PP + PV and PV groups, it was higher, at 13.8 and 14.6, respectively (*p*-value = 0.007).

At baseline, the DLQI was significantly higher in the PP group (18) than in the PP + PV (16.5) and PV (11.2) groups (*p* = 0.036).

The Visual Analogue Scale (VAS) scores for itch were similar across the three groups: 6.2 for the pure PP group, 6.4 for the PP + PV group, and 6.4 for the PV group (*p* = 0.862).

#### 3.3.1. Efficacy: Mean % PASI/DLQI/VAS Reduction and Response Rates

**Figure 1:**
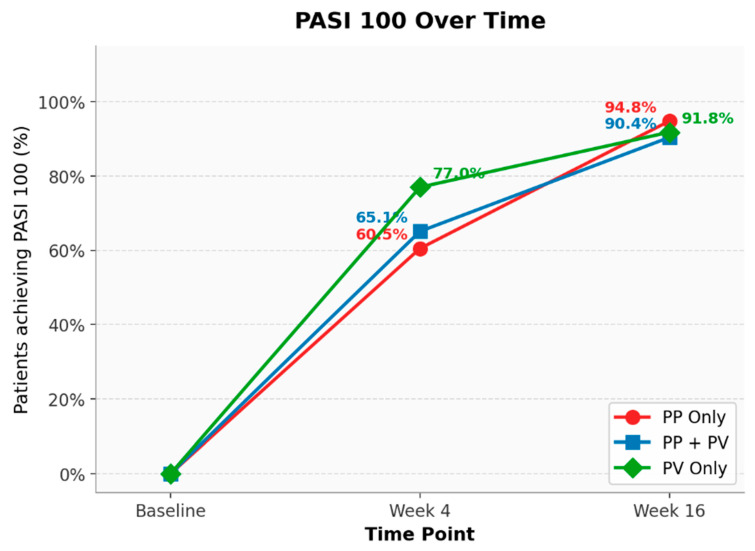
Patients achieving PASI 100 over time (week 16) (PP, palmoplantar psoriasis; PV, psoriasis vulgaris).

The mean % PASI reduction at Week 4 was 60.5% in the PP only group, 65.1% in the PP + PV group, and 77.0% in the PV only group (*p* = 0.027), increasing to 94.8%, 90.4%, and 91.8% at week 16 (*p* = 0.011). When expressed as conventional PASI100 (proportion of patients achieving PASI = 0), response rates at week 4 were 26.7% (4/15), 13.6% (3/22), and 30.0% (3/10) (*p* = 0.477), rising to 73.3% (11/15), 68.2% (15/22), and 70.0% (7/10) at week 16 (*p* = 0.945). Between-group differences were not statistically significant at either timepoint (Table 3, Figure 1).

**Figure 2:**
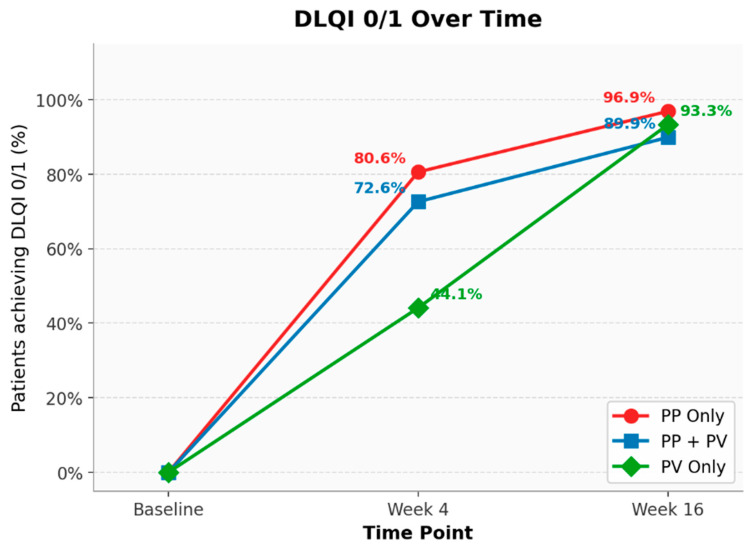
Patients achieving DLQI 0/1 over time (week 16) (PP, palmoplantar psoriasis; PV, psoriasis vulgaris; DLQI, Dermatology Life Quality Index).

The mean % DLQI reduction at week 4 was 80.6% in the PP only group, 72.6% in the PP + PV group, and 44.1% in the PV only group (*p* = 0.521), increasing to 96.9%, 89.9%, and 93.3% at week 16 (*p* = 0.855). The proportion of patients achieving DLQI 0/1 at week 4 was 20.0% (3/15), 45.5% (10/22), and 30.0% (3/10) (*p* = 0.264), rising to 80.0% (12/15), 81.8% (18/22), and 80.0% (8/10) at week 16 (*p* = 0.988). Between-group differences were not statistically significant at either timepoint (Table 4, Figure 2).

**Figure 3:**
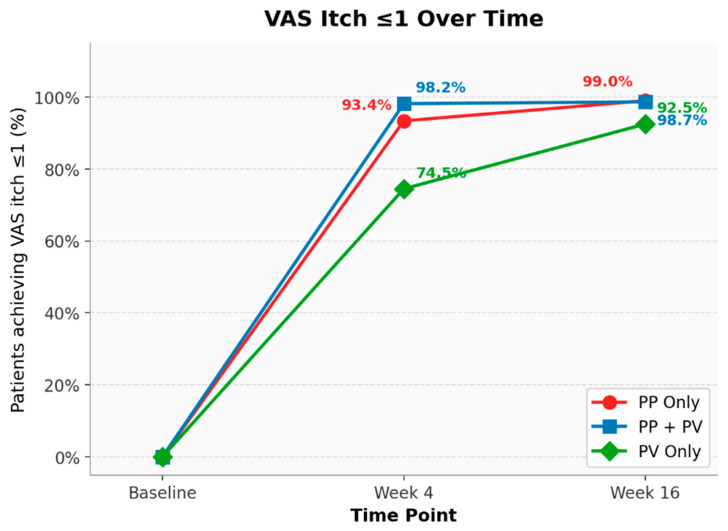
Patients achieving VAS itch 0/1 over time (Week 16) (PP, palmoplantar psoriasis; PV, psoriasis vulgaris; VAS, Visual Analogue Scale).

The mean % VAS itch reduction at Week 4 was 93.4% in the PP only group, 98.2% in the PP + PV group, and 74.5% in the PV only group, with a statistically significant between-group difference (*p* = 0.012). At week 16, the mean % VAS reductions were 99.0%, 98.7%, and 92.5% (*p* = 0.787). The proportions of patients achieving VAS itch ≤1 at week 4 were 86.7% (13/15), 95.5% (21/22), and 60.0% (6/10) (*p* = 0.032), rising to 100.0% (15/15), 95.5% (21/22), and 90.0% (9/10) at week 16 (*p* = 0.477). Between-group differences at week 16 were not statistically significant (Table 5, Figure 3.)

#### 3.3.2. Efficacy (PASI 100) According to the Bio-Naïve or Bio Experience Status

When stratifying by prior biologic exposure, a higher rate of PASI 100 at week 16 was observed among bio-naïve compared to bio-experienced patients in the PV group (100.0% vs. 57.1%; Fisher’s exact test: *p* = 0.475) and in the PP + PV group (75.0% vs. 64.3%; *p* = 0.876), although neither difference reached statistical significance, likely due to small sample sizes. Notably, in the PP group, the pattern was reversed: bio-naïve and bio-experienced patients achieved similar PASI 100 rates at week 16 (57.1% vs. 87.5%; Fisher’s exact test: *p* = 0.282). All comparisons should be considered exploratory given the limited sample sizes, particularly in the bio-experienced subgroups.

### 3.4. Comparison of PASI, DLQI, and VAS Itch Scores at Weeks 4 and 16 for All Three Groups

A statistically significant improvement in PASI was observed from week 4 to week 16 in the PP group (mean PASI 4.3 ± 3.7 at week 4 vs. 0.6 ± 1.1 at week 16; Wilcoxon signed-rank test: *p* = 0.003), the PP + PV group (4.8 ± 4.8 vs. 0.9 ± 1.5; *p* < 0.001), and the PV group (4.0 ± 4.4 vs. 1.0 ± 1.9; *p* = 0.011).

Regarding DLQI, all three groups demonstrated further reductions at week 16 compared to week 4: PP group (mean DLQI 4.5 ± 3.9 at week 4 vs. 0.6 ± 1.3 at week 16; Wilcoxon: *p* = 0.009), PP + PV group (4.3 ± 5.2 vs. 1.2 ± 2.9; *p* = 0.001), and PV group (4.5 ± 4.2 vs. 0.6 ± 1.1; *p* = 0.036). Conversely, no statistically significant differences in the VAS for itch were observed across the three subgroups. The mean absolute changes from baseline to week 16 were as follows: PASI −11.1 ± 7.8 (PP), −12.9 ± 8.2 (PP + PV), −13.6 ± 8.8 (PV); DLQI −17.4 ± 8.7, −15.3 ± 7.8, −10.6 ± 6.2; VAS itch −6.1 ± 4.2, −6.3 ± 3.0, −6.1 ± 3.3, respectively.

### 3.5. Safety

No serious adverse events (SAEs), no severe infections, and no treatment discontinuations due to adverse events were observed.

Reported adverse events were mild and consistent with the known safety profile of bimekizumab. The most frequently reported adverse events were upper respiratory tract infections/nasopharyngitis, occurring in four patients (8.5%). Mild oral candidiasis was observed in two patients (4.3%) and was successfully managed with topical antifungal treatment without treatment interruption. Injection-site reactions were reported in two patients (4.3%) and were transient and self-limiting. Sporadic cases of headache and fatigue were reported in one patient each (2.1%).

Overall, bimekizumab was well tolerated in this real-world cohort of patients with plaque psoriasis with or without palmoplantar involvement.

### 3.6. Limitations

This study has several limitations. First, the small sample size (n = 47) and unequal group sizes preclude the application of adequately powered multivariable models adjusted for confounders such as BMI and baseline disease severity, both of which differed significantly across groups. Second, the retrospective design and requirement for complete follow-up data may have introduced selection bias toward more adherent and responsive patients. Third, the multicenter setting introduces potential center-level clustering effects that could not be formally accounted for. Fourth, all *p*-values reported are nominal and exploratory; no correction for multiple comparisons was applied, and no confirmatory conclusions should be drawn pending validation in larger prospective cohorts.

Another limitation of the manuscript could be the absence of data about durability beyond 16 weeks. We would like to perform further study on that. However, at the moment, we consider these data described interesting and very useful.

## 4. Discussion

Palmoplantar psoriasis (PP) can occur as an isolated form in 3–4% of cases or as a localized variant of extensive psoriasis vulgaris (PV) [11]. Although limited in extent, PP significantly impairs the physical and functional quality of life for individuals affected by it. Our cohort data support this observation, showing that the PP group had higher Dermatology Life Quality Index (DLQI) scores than the comparative group [5].

Currently, topical treatments and traditional systemic therapies are considered the first-line options for patients with exclusive PP [12]. However, these treatments may not always be fully effective, and there is a lack of randomized controlled trials specifically addressing this population [10]. Many authors suggest that a body surface area (BSA) of less than 10% should not preclude the consideration of biological therapies for patients with PP [1,12,13]. Given the crucial roles of IL-17 and IL-23 in the pathogenesis of PP, targeting these cytokines may offer therapeutic benefits [12]. Research has demonstrated that IL-17 is highly expressed in PP lesions, while IL-23 plays a vital role in the differentiation and maintenance of T helper 17 cells, which produce IL-17 and sustain the disease [12,13].

Among the IL-17 inhibitors, BKZ has a unique ability to target both IL-17A and IL-17F. According to a recent network meta-analysis by Spencer et al., BKZ is one of the most effective therapies for treating PP [6]. Additionally, Merola et al. demonstrated a rapid clearance at week 16 in 87.4% of patients treated with BKZ, which was maintained through the end of the second year, from 88.3% at year 1 to 89.8% at year 2 [14].

Bianco et al. conducted a real-word, single-center study on effectiveness of bimekizumab for plaque psoriasis involving difficult-to-treat areas, and results are in line with ours [15].

The relevance of the dual blockade of IL17A and IL17F may rely on the synergist role of IL17F and TNF in dermal fibroblasts, not only keratinocytes, contributing to chronic inflammation [16]. Results of transcriptional profiling from Svraka et al. may explain the rapid action of bimekizumab in PPP due to its dual inhibition of IL17A and IL17 F [16].

However, these data do not differentiate between patients with only palmoplantar psoriasis and those with both PP and PV, which is the primary purpose of our study. This distinction enables us to evaluate the efficacy of BKZ across various patient groups. Our findings reveal some interesting differences in baseline characteristics. Although no significant differences in age or sex were observed across the three groups, patients with only PV had a higher body mass index (BMI) than those in the other groups. Previous studies have suggested that visceral fat can promote the Koebner phenomenon in psoriasis, hence linking the high BMI to the presence of psoriasis on palmo-plantar areas of patients due to mechanical stress [17].

Although the Fisher‘s exact test did not reach statistical significance, a numerically higher proportion of males was observed in the PP-only group (73.3%) compared to the PP + PV (54.5%) and PV (50.0%) groups; this trend warrants further investigation in larger cohorts. Regarding the DLQI, those with exclusive PP reported higher scores. This type of psoriasis limits even minimal daily activities, adversely affecting quality of life more than psoriasis that is covered by clothing [18]. As expected, the baseline PASI score was higher in patients with PV or with both PV and PP than in those with only PP. Interestingly, there was no statistically significant difference in the Visual Analogue Scale (VAS) itch score among the three groups.

Regarding PASI 100 efficacy, in line with Merola et al.’s pooled analysis [14], we observed a favorable reduction in the PASI, DLQI, and VAS itch. Notably, all parameters showed a statistically significant decrease from baseline to week 4 and were maintained; the percentage of patients reaching complete clearance of psoriasis further increased at week 16.

**Figure 4:**
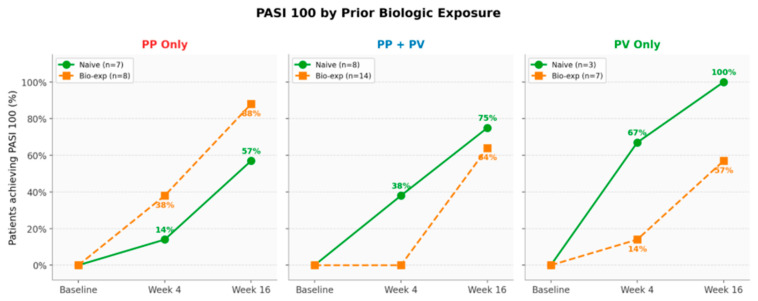
Patients achieving PASI 100 over time (week 16) comparing bio-naïve and bio-experienced in all subgroups (PP, palmoplantar psoriasis; PV, psoriasis vulgaris, Bio-exp, bio-experienced).

Among patients receiving prior therapy, those in both the PV group and the PV + PP group achieved PASI100 at week 16 more often than those in the PP group (Figure 4). Nevertheless, when considering the PP group, multi-failure patients reached PASI100 already at week 4, suggesting that the disease might have relied heavily on IL17A and IL17F as drivers of inflammation [7]. It is worth noticing that these results might be also biased by the small number of patients in this exploratory analysis.

Given the small sample size and the exploratory nature of this analysis, no definitive conclusions can be drawn regarding differences between patients previously exposed to 1 versus ≥2 biologics. However, our findings appear consistent with previous literature reporting reduced early super-response rates among multi-failure patients [19]. In fact, Esposito et al. have shown that naive status or failure of less than two biologics was correlated with a higher probability of reaching PASI100 in the short term [19]. Similarly, Fratton et al. conducted a multivariate analysis of baseline characteristics associated with an early clinical response and found that multifailure patients were less likely to be early responders to the drug [20]. Taken together, these data support the earlier use of BKZ, to maximize its efficacy in clinical practice.

Several other studies have analyzed the efficacy of BKZ in patients with psoriasis in high-impact areas. Campione et al. investigated the efficacy of BKZ in patients with nail psoriasis [21], while Orsini et al. studied the efficacy of BKZ in patients with genital psoriasis [22]. In both studies, treatment with BKZ was effective and allowed the majority of treated patients to achieve complete clearance of interested areas [21,22]. Interestingly, previous studies suggested that the presence of palmoplantar psoriasis in patients might correlate with a decreased likelihood of achieving PASI100 in the short term for BKZ [23] and secukinumab [24]. Our paper explores how this effect might be true for patients that exhibit psoriasis only in the PP area since the magnitude of the response in the first weeks of treatment has been higher in the PV and PV + PP groups compared to patients in the PP group. Trovato et al. have previously described a favorable effect of treatment with BKZ in a patient with PP refractory to adalimumab [25]. Taken together, our work is consistent with previous report and support the efficacy in clinical practice of BKZ for the treatment of patients with psoriasis in high impact areas, such as the palmoplantar area.

## 5. Conclusions

BKZ demonstrated strong efficacy in both patients with psoriasis vulgaris and palmoplantar involvement, reducing PASI, DLQI, and VAS itch scores from week 4 to week 16. Although the study has a limitation due to its small sample size, it is one of the few comparing patients with only palmoplantar psoriasis to patients with palmoplantar lesions associated with psoriasis vulgaris (PV) and patients with only PV.

According to our findings, we believe that BKZ should be considered for a rapid and effective response. Larger studies are necessary to confirm these findings.

## Figures and Tables

**Table 1:** Epidemiological characteristics of patients enrolled in the study (SD, standard deviation; IQR, interquartile range).

Variable	Value (n = 47)
Patients, n	47
Sex, n (%)	Female: 40.4% (n = 19), Male: 59.6% (n = 28)
Age (years) mean ± SD (median; IQR)	58.2 ± 11.6 (59.0; 52.5–65.0)
BMI (kg/m^2^) mean ± SD (median; IQR)	27.4 ± 4.9 (26.1; 24.0–30.3)
Disease duration (years) mean ± SD (median; IQR)	14.4 ± 14.2 (9.5; 3.0–23.0)
Baseline PASI mean ± SD (median; IQR)	13.3 ± 8.1 (12.0; 8.9–20.0)
Baseline DLQI mean ± SD (median; IQR)	15.9 ± 8.0 (17.0; 12.0–22.0)
Baseline VAS itch mean ± SD (median; IQR)	6.3 ± 3.4 (7.5; 5.0–8.0)

**Table 2:** Epidemiological characteristics of patients enrolled in the study divided in the two cohorts: patients (PP only, PP + PV) and comparative group (PV only) (SD, standard deviation; IQR, interquartile range; BMI, body mass index; PASI, Psoriasis Area and Severity Index; DLQI, Dermatology Life Quality Index; Bio-exp, bio-experienced).

Variable	PP Only (n = 15)	PP + PV (n = 22)	PV Only (n = 10)	*p*-Value
Sex (% male)	73.3% (n = 11)	54.5% (n = 12)	50.0% (n = 5)	0.408
Age (years) mean ± SD median (IQR)	57.3 ± 14.5 60.0 (55.0–65.0)	58.4 ± 10.0 59.5 (51.2–65.0)	60.7 ± 10.6 58.5 (54.0–69.2)	0.969
BMI (kg/m^2^) mean ± SD median (IQR)	26.8 ± 3.7 27.1 (23.6–30.0)	27.1 ± 5.6 25.8 (23.7–28.7)	30.6 ± 4.9 29.0 (25.1–33.1)	0.045
Disease duration (years) mean ± SD median (IQR)	12.9 ± 11.5 9.5 (3.2–20.8)	13.9 ± 13.9 9.5 (2.2–19.2)	19.9 ± 18.9 14.5 (2.2–38.2)	0.844
Baseline PASI mean ± SD median (IQR)	7.7 ± 3.1 7.5 (5.6–10.5)	13.8 ± 8.2 12.1 (8.9–19.2)	14.6 ± 8.2 12.2 (12.0–18.6)	0.007
Baseline DLQI mean ± SD median (IQR)	18.0 ± 9.0 19.0 (16.0–23.0)	16.5 ± 7.6 16.5 (12.0–22.8)	11.2 ± 6.1 13.0 (9.0–14.8)	0.036
Baseline VAS itch mean ± SD median (IQR)	6.2 ± 4.2 8.0 (2.0–10.0)	6.4 ± 3.0 7.0 (5.2–8.0)	6.4 ± 2.9 8.0 (5.0–8.0)	0.862
Bio-naïve, n (%)	7 (46.7%)	8 (36.4%)	3 (30.0%)	n/a
Bio-exp. 1 biologic, n (%)	6 (40.0%)	11 (50.0%)	4 (40.0%)	n/a
Bio-exp. ≥ 2 biologics, n (%)	2 (13.3%)	3 (13.6%)	3 (30.0%)	n/a

**Table 3:** PASI sub-analysis by groups (PASI, Psoriasis Area and Severity Index; W, week; PP, palmoplantar psoriasis; PV, psoriasis vulgaris).

	PP Only (n = 15)	PP + PV (n = 22)	PV Only (n = 10)	*p*-Value
Mean % PASI reduction from baseline				
Mean % PASI reduction at Week 4	60.5%	65.1%	77.0%	0.027
Mean % PASI reduction at Week 16	94.8%	90.4%	91.8%	0.011
PASI100—conventional complete clearance (PASI = 0) → NEW ROWS				
PASI = 0 (PASI100) at Week 4, n (%)	4 (26.7%)	3 (13.6%)	3 (30.0%)	0.477
PASI = 0 (PASI100) at Week 16, n (%)	11 (73.3%)	15 (68.2%)	7 (70.0%)	0.945
PASI75 → EXISTING				
PASI75 at Week 4, n (%)	4 (26.7%)	9 (40.9%)	6 (60.0%)	0.250
PASI75 at Week 16, n (%)	12 (80.0%)	20 (90.9%)	9 (90.0%)	0.594
Absolute scores				
PASI mean ± SD at Week 4	4.3 ± 3.7	4.8 ± 4.8	4.0 ± 4.4	0.830
PASI median (IQR) at Week 4	4.0 (1.0–6.5)	3.0 (1.0–6.9)	2.0 (0.5–7.8)	—
PASI mean ± SD at Week 16	0.6 ± 1.1	0.9 ± 1.5	1.0 ± 1.9	0.909
PASI median (IQR) at Week 16	0.0 (0.0–1.0)	0.0 (0.0–2.0)	0.0 (0.0–1.5)	—
Change from baseline				
Mean change BL → W16, Δ (mean ± SD)	−11.1 ± 7.8	−12.9 ± 8.2	−13.6 ± 8.8	—
W4 → W16 Wilcoxon *p*	0.003	<0.001	0.188	—

**Table 4:** DLQI 0/1 sub-analysis by groups (DLQI, Dermatology Life Quality Index).

	PP Only (n = 15)	PP + PV (n = 22)	PV Only (n = 10)	*p*-Value
Mean % DLQI reduction from baseline → EXISTING ROWS (label corrected + *p*-value corrected)				
Mean % DLQI reduction at Week 4	80.6%	72.6%	44.1%	0.0513
Mean % DLQI reduction at Week 16	96.9%	89.9%	93.3%	0.0672
DLQI 0/1—conventional response → NEW ROWS				
DLQI 0/1 at Week 4, n (%)	3 (20.0%)	10 (45.5%)	3 (30.0%)	0.264
DLQI 0/1 at Week 16, n (%)	12 (80.0%)	18 (81.8%)	8 (80.0%)	0.988
Absolute scores → EXISTING (add median rows; correct Wilcoxon *p*-values)				
DLQI mean ± SD at Week 4	4.5 ± 3.9	4.3 ± 5.2	4.5 ± 4.2	0.773
DLQI median (IQR) at Week 4	4.0 (2.0–5.5)	3.0 (0.0–5.5)	3.5 (1.2–7.2)	—
DLQI mean ± SD at Week 16	0.6 ± 1.3	1.2 ± 2.9	0.6 ± 1.1	0.880
DLQI median (IQR) at Week 16	0.0 (0.0–0.0)	0.0 (0.0–0.8)	0.0 (0.0–0.8)	—
Change from baseline → NEW ROWS				
Mean change BL → W16, Δ (mean ± SD)	−17.4 ± 8.7	−15.3 ± 7.8	−10.6 ± 6.2	—
W4 → W16 Wilcoxon *p*	0.003	0.001	0.016	—

**Table 5:** VAS itch 0/1 sub-analysis by groups (PP, palmoplantar psoriasis; PV, psoriasis vulgaris; VAS, Visual Analogue Scale; W, week; SD, standard deviation).

	PP Only (n = 15)	PP + PV (n = 22)	PV Only (n = 10)	*p*-Value
Mean % VAS itch reduction from baseline				
Mean % VAS reduction at Week 4	93.4%	98.2%	74.5%	0.012
Mean % VAS reduction at Week 16	99.0%	98.7%	92.5%	0.787
VAS itch ≤ 1				
VAS itch ≤ 1 at Week 4, n (%)	13 (86.7%)	21 (95.5%)	6 (60.0%)	0.032
VAS itch ≤ 1 at Week 16, n (%)	15 (100.0%)	21 (95.5%)	9 (90.0%)	0.477
Absolute scores				
VAS mean ± SD at Week 4	0.4 ± 1.1	0.2 ± 0.5	1.3 ± 1.6	0.035
VAS median (IQR) at Week 4	0.0 (0.0–0.0)	0.0 (0.0–0.0)	0.5 (0.0–2.0)	—
VAS mean ± SD at Week 16	0.1 ± 0.3	0.1 ± 0.4	0.3 ± 0.9	0.823
VAS median (IQR) at Week 16	0.0 (0.0–0.0)	0.0 (0.0–0.0)	0.0 (0.0–0.0)	—
Change from baseline				
Mean change BL → W16, Δ (mean ± SD)	−6.1 ± 4.2	−6.3 ± 3.0	−6.1 ± 3.3	—
W4 → W16 Wilcoxon *p*	0.180	0.578	0.063	—

## Data Availability

Data are available on request from the authors.

## Abbreviations

The following abbreviations are used in this manuscript:

BKZ	Bimekizumab
BMI	Body Mass Index
BSA	Body Surface Area
DLQI	Dermatology Life Quality Index
IQR	Interquartile Range
PASI	Psoriasis Area and Severity Index
PP	Palmoplantar psoriasis
PV	Psoriasis vulgaris
SD	Standard Deviation
VAS	Visual Analogue Scale
W4	Week 4
W16	Week 16
